# Mechanistic Pathways in Cyanide-Mediated Benzoin Condensation: A Comprehensive Electron Localisation Function (ELF) and Catastrophe Theory Analysis of the Umpolung Reaction

**DOI:** 10.3390/molecules30020378

**Published:** 2025-01-17

**Authors:** Michal Michalski, Slawomir Berski

**Affiliations:** 1 Centre of New Technologies, University of Warsaw, 02-097 Warsaw, Poland; m.michalski@cent.uw.edu.pl; 2 Faculty of Chemistry, University of Wroclaw, 50-383 Wroclaw, Poland

**Keywords:** umpolung reaction, benzoin condensation, BET, ELF

## Abstract

This research investigates the mechanism of the cyanide-type umpolung reaction in benzoin condensation using topological analysis of ELF and catastrophe theory. The study achieves a comprehensive understanding of the evolution of chemical bonds and non-bonding electron density in the reaction of benzaldehyde and cyanide ions. The results reveal that the reaction proceeds through five transition state structures, with the formation of Lapworth’s cyanohydrin being the rate-determining step. The study characterises topological catastrophes in the evolution of the ELF field and provides a detailed description of the evolution of electron density in the mechanism of the reaction. An in-depth analysis of ELF catastrophes confirms the well-established Lapworth mechanism.

## 1. Introduction

In 1903, Lapworth provided a pioneering account of the mechanism underlying the cyanide-catalysed benzoin condensation [1], which has since been widely studied in the field of organic chemistry [2,3,4,5,6]. This reaction belongs to the class of umpolung-type reactions, where the polarity of the functional group is reversed due to its chemical modification [7]. The proposed mechanism involves the formation of a cyanohydrin intermediate, which serves as a crucial metastable state in facilitating the reaction. The benzoin condensation is a dimerisation reaction involving two aldehyde molecules, where one aldehyde donates a hydrogen atom and the other accepts it. Notably, this reaction proceeds without generating a small byproduct molecule. Wiberg in his pioneering investigation observed inconsistencies with the classic Lapworth mechanism when using a 66% ethanol–water solvent, suggesting deviations from the expected mechanism [8,9]. Breslow and colleagues examined the influence of salts on the hydrophobic acceleration and anti-hydrophobic effects, finding that the reaction rate in dimethyl sulfoxide (DMSO) is approximately 50 times greater than in water without additives and roughly 10,000 times greater than in ethanol [10,11]. While protonic solvents like methanol, ethanol, and water are commonly employed, DMSO stands out as an aprotic solvent that does not promote proton transfer or intermediate formation, potentially explaining the significantly enhanced reaction rate observed in this medium [12]. This highlights the need for further investigation to gain a deeper understanding of the umpolung mechanism.

In this work, a theoretical study of the Lapworth mechanism of cyanide-catalysed benzoin condensation in the gas phase was conducted using Density Functional Theory (DFT) and Bonding Evolution Theory (BET) [13] methods. The BET is based on the topological analysis of the electron localisation function (ELF) developed by Silvi and Savin [14,15] and on Thom’s catastrophe theory [16]. This method has been employed in previous studies to analyse the mechanisms of organic reactions [17,18,19,20,21] and offers an attractive alternative to Hilbert space descriptions. The electronic structure of the reaction can be characterised through ELF topology, with ELF regions, or basins, classified into core basins (C(X)) and valence basins (V(A,B,…)), each associated with a local maximum or attractor in the ELF field. Two primary types of valence basins can be identified: monosynaptic basins, V(A), representing lone pairs in Lewis structures, and disynaptic basins, V(A,B), which denote covalent bonds between atoms A and B [22,23]. As the reaction progresses, structural changes in the ELF field can be observed, with attractors and critical points (CPs) undergoing abrupt transitions called catastrophes. Three main types of topological catastrophes—fold, cusp, and elliptic umbilic—describe unique CP transformations. To qualitatively track the ELF field’s evolution, Hausdorff distances between consecutive points along the intrinsic reaction coordinate (IRC) path can be calculated [24,25] to capture the ELF field’s stepwise progression. In this study, we aim to answer the following questions: (1) What is the order of bond breaking and formation from the BET perspective? (2) What is the ELF-topological depiction of polarity reversal on the carbon atom, the core of the “umpolung” type reaction? (3) Does the representation of the reaction mechanism based on ELF topological analysis and catastrophe theory align with the classical Lewis structure representation of the mechanism? We believe the presented study provides a deeper understanding of the mechanism of the umpolung reaction.

## 2. Results and Discussion

In this work, we present a theoretical study of the cyanide-catalysed benzoin condensation, which belongs to the class of umpolung reactions. The study is divided into two parts: (I) an energy analysis of stationary points on PES and (II) an analysis of the bond-breaking and bond-creating events using BET and Hausdorff distances. In the first part, we employ DFT to determine thermodynamics for the reaction. In the second part, we use the topological analysis of ELF to investigate the bond-breaking and bond-creating events during the reaction. We use the Hausdorff distances to quantitatively evaluate the changes in the topology of the electron density during the reaction. The proposed approach of combining energy analysis and ELF analysis allows for a comprehensive examination of the reaction mechanism, providing insights into the changes in the electronic structure and the topology of the electron density during the reaction.

### 2.1. The Energetic of the Reaction and Geometrical Analysis

**Figure 1:**
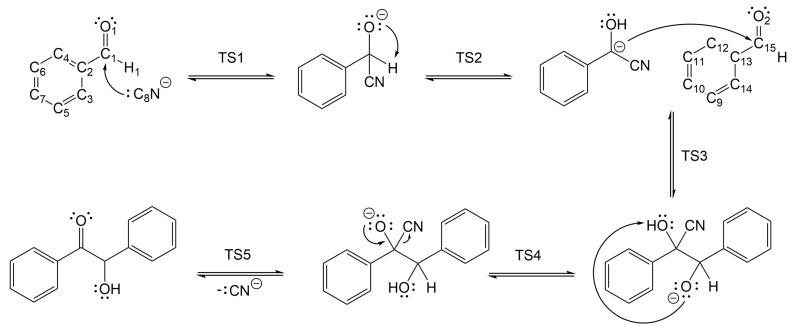
The Lapworth mechanism of the cyanide-catalysed benzoin condensation.

**Figure 2:**
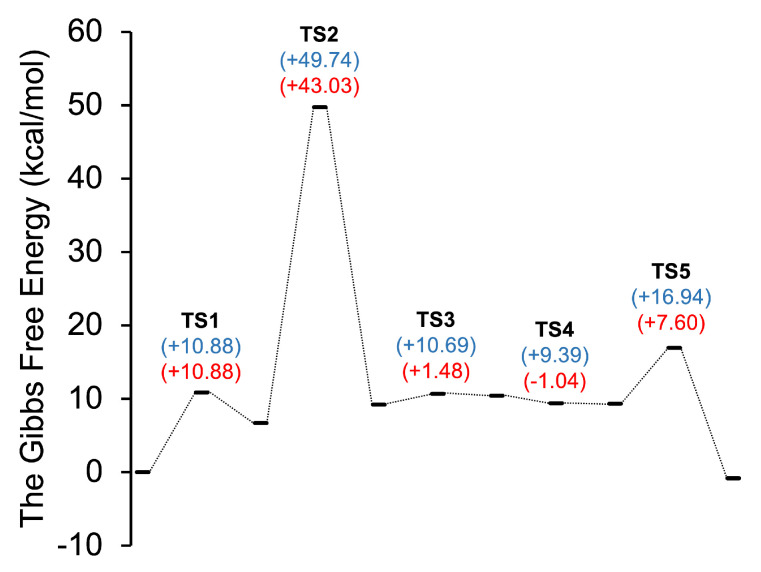
The Gibbs free energy of activation barriers and relative Gibbs free energies for umpolung transition states (TSs), with blue values representing relative energies and red values indicating activation energies from the reagent side, $\Delta G_{a}\left(I\right)$.

**Figure 3:**
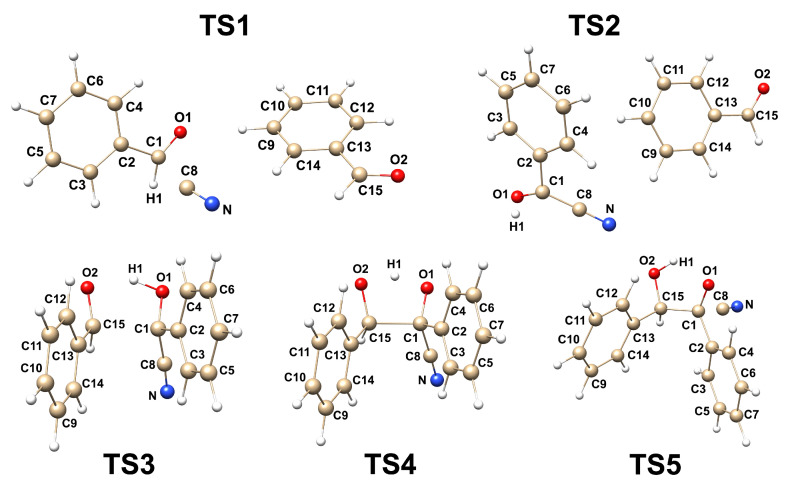
Optimised transition state (TS) structures for all steps of the umpolung reaction at the DFT(B3LYP)/6-311++G(d,p) computational level.

**Figure 4:**
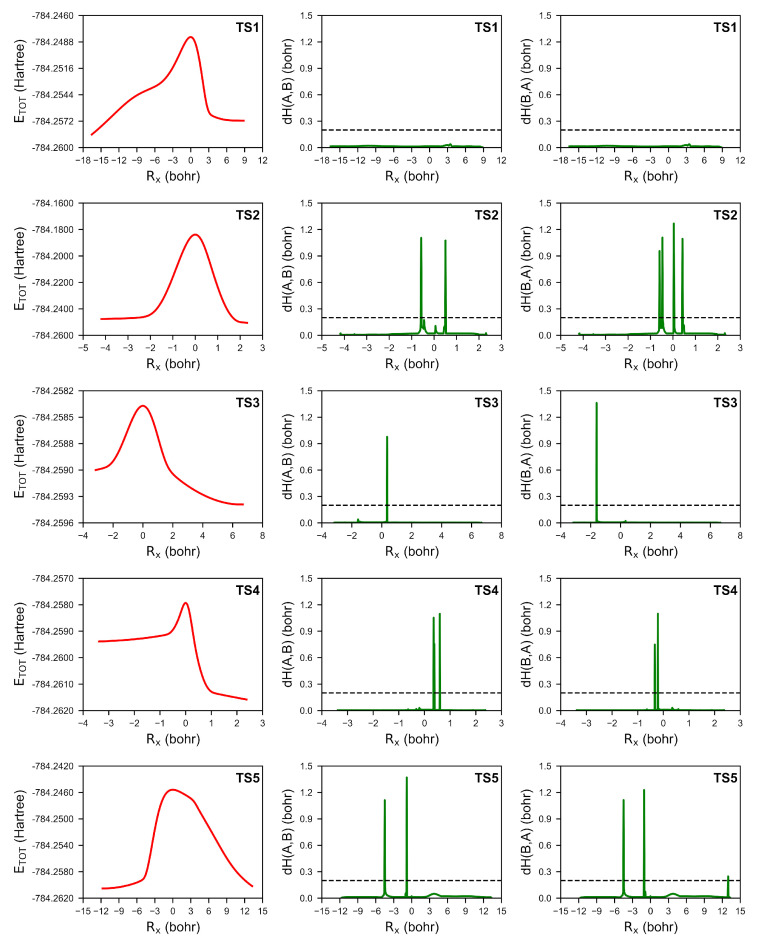
The evolution of the total energy (red lines), $E_{\mathrm{TOT}}$, and both Hausdorff distances (green lines), dH(A,B) and dH(B,A), for the umpolung reaction as a function of the IRC coordinate. The peaks above the dashed line (dH = 0.2) indicate significant topological changes in the ELF map.

The mechanism of the cyanide-catalysed benzoin condensation was formally studied and presented in Figure 1. An analysis of PES revealed that the reaction proceeds through five transition state structures in the gas phase at 298.15 K in the absence of solvent assistance. The Gibbs free energy barriers were depicted in Figure 2, with the corresponding transition state structures shown in Figure 3, and the energy profiles illustrated in Figure 4.

In the first step (TS1), the nucleophile cyanide ion attacks the carbon atom of the C=O group in benzaldehyde. As a result, an electron pair from the C1=O1 bond is transferred to the oxygen atom, leading to the formation of a negatively charged oxygen atom. The second step (TS2) is associated with the migration of a hydrogen atom to the O1 atom, resulting from the breaking of the C1-H1 bond. During this step, an electron pair from the O1 atom is transferred toward the migrating H1 atom, which now bears a positive charge. This step also results in a change in the hybridisation of the C1 atom from ${\mathrm{sp}}^{3}$ to ${\mathrm{sp}}^{2}$, as well as a reversal of the polarity of the carbonyl group. The product of this step, known in the literature as the Lapworth’s cyanohydrin intermediate [12,26], serves as the key intermediate in the subsequent steps of the reaction. In the third step (TS3), due to the negative charge on the C1 atom, Lapworth’s intermediate reacts with benzaldehyde in a second nucleophilic addition, leading to the formation of a new C1-C15 bond. An electron pair from the C15=O2 group is transferred to the oxygen atom, resulting in the formation of a negatively charged oxygen atom. The fourth step (TS4) involves the transfer of a single electron pair from the O2 atom to the H1 atom, resulting in a migration of the H1 proton and the formation of a new O-H bond. This step marks the formation of the final product, benzoin. Lastly, in the fifth step (TS5), a single electron pair from the O1 atom is moved towards the C=O bond, while another electron pair from the C1-C8 bond is moved to the carbon atom in the -CN group. This results in the release of the cyanide ion and the formation of benzoin as the final product.

The Gibbs free energies of activation, $\Delta G_{a}\left(I,\mathrm{II}\right)$, associated with the transition states TS1-TS5 were calculated using the B3LYP electron density functional and are presented in Table 1. The values of $\Delta G_{a}\left(I,\mathrm{II}\right)$ were determined for both the reactant side, $\Delta G_{a}\left(I\right)$, and the product side, $\Delta G_{a}\left(\mathrm{II}\right)$, of each transition state.

The first step (TS1) is characterised by the nucleophilic attack of the cyanide ion on the carbon atom of the carbonyl group in benzaldehyde. The imaginary frequency of TS1 was calculated to be 218.860i ${\mathrm{cm}}^{-1}$, and its vibrational vector corresponds to the formation of the C1-C8 bond. The distance of the C1-C8 bond in the pre-reaction and post-reaction complexes was found to be 4.260 and 1.531 Å, respectively. The activation energy associated with this step, $\Delta G_{a}\left(I\right)$, was calculated to be 10.88 kcal/mol. This value is 6.71 kcal/mol higher than the corresponding value for the product. The $\Delta G_{r}$ was found to be positive in the gas phase, indicating that the reaction is thermodynamically not spontaneous.

The second step (TS2) is characterised by the transfer of a proton between the C1 and O1 atoms, resulting in the formation of the Lapworth’s cyanohydrin intermediate. This step is associated with a change in the hybridisation of the C1 atom from ${\mathrm{sp}}^{3}$ to ${\mathrm{sp}}^{2}$ and is accompanied by an elongation of the C1-O1 bond length from 1.328 Å to 1.422 Å, which is within the typical range of single C-O interactions [27]. The $\Delta G_{a}\left(I\right)$, $\Delta G_{a}\left(\mathrm{II}\right)$, and $\Delta G_{r}$ associated with this step were calculated to be 43.03, 40.53, and 2.50 kcal/mol, respectively. These values indicate that the energy barriers for this step are significantly higher than those for TS1, making it the rate-determining step of the reaction. It is noteworthy that this step has the highest energy activation barrier among all the steps, making it the most energetically difficult step in the reaction mechanism. This problem was investigated using DFT calculations with the CPCM solvation model (DMSO, water, ethanol, methanol) [12]. No significant impact on the barrier height was observed when considering solvent effects. However, a proton transfer system assisted by methanol was simulated, showing a decrease in the barrier height by approximately 25 kcal/mol. We presume that the step leading to the formation of the cyanohydrin intermediate may proceed via a solvent-assisted proton shift transition state in protonic solvent [12].

The reaction of the Lapworth’s intermediate with benzaldehyde leads to the formation of a C1-C15 bond. The C1-C15 bond length in the pre-reaction, transition state (TS3), and post-reaction complexes are 2.480, 2.091, and 1.760 Å, respectively. The imaginary frequency of TS3 is associated with the formation of the C1-C15 bond. The activation energy of the step, as calculated using the B3LYP method, is relatively low at 1.48 kcal/mol when considering the energy barrier from the side of the reagents. However, the energy barrier from the side of the product is even lower at 0.26 kcal/mol. Thus, the Gibbs free energy of reaction is positive, indicating that this process is not spontaneous.

In the fourth step (TS4), a proton transfer occurs between the O1 and H1 atoms. The associated activation energy, as calculated using B3LYP, is −1.04 kcal/mol. This value is unphysical and negative, which is indicative of a barrier-less reaction. Analysis of PES reveals that the C-O distance between the O1 and H1 atoms elongates from 1.186 to 1.242 Å during this step. The negative sign of the activation energy is attributed to the fact that the zero-point vibrational energy of the pre-reaction complex is larger than that of the transition state, 143.627 kcal/mol and 141.667 kcal/mol, respectively. To further verify these results, the activation energies were also calculated using additional DFT functionals and basis sets, as presented in Table 2. The results obtained using these alternative methods also indicated a negative activation energy, consistent with the literature on barrier-less reactions [28,29,30].

In the final step (TS5), the mechanism of benzoin condensation proceeds via the elimination of the -CN group. The vibrational frequency of TS5 has been determined to be 129.984i ${\mathrm{cm}}^{-1}$, corresponding to the breaking of the C1-C8 bond. The $\Delta G_{a}\left(I\right)$ and $\Delta G_{r}$ associated with this step have been calculated to be 7.60 and 17.77 kcal/mol, respectively. These values indicate that at room temperature and in the gas phase, this process is thermodynamically spontaneous.

The overall $\Delta G_{r}$ value for the umpolung reaction is −0.83 kcal/mol, indicating that the reaction is thermodynamically spontaneous under the conditions considered. This overall reaction character aligns with the energetic profile, as each step described earlier contributes to the overall process. It is important to note that these calculations were performed in the gas phase and thus do not account for potential solvent effects. The solvent can play a crucial role in stabilising reaction intermediates and transition states, which may further reduce the activation barriers and enhance the thermodynamic favorability of the reaction.

### 2.2. The Mechanism of the Reaction—BET Analysis

The Hausdorff distances for TS1 were found to exhibit a flat baseline with no strong peaks (as seen in Figure 4). This indicates that no catastrophes related to the evolution of the ELF field attractors were observed. However, at a value of $R_{x}$ = −0.137 Bohr, a change in V(C8) basin synapticity to V(C1,C8) was observed, signalling the formation of a C1-C8 bond with a population of 2.58e. The V(C1,C8) attractor was found to be localised in the position of V(C8) without any additional changes in the ELF field. A topological analysis of the ELF for the post-reaction complex revealed a flux of electron density from V(O1,C1) to $V_{1}$(O1), which is in good agreement with the elongation of the C1-O1 bond length. The C1-C8 and C8-N bonds were found to be described by V(C1,C8) and V(C8,N) basins with populations of 2.24e and 4.33e, respectively.

**Figure 5:**
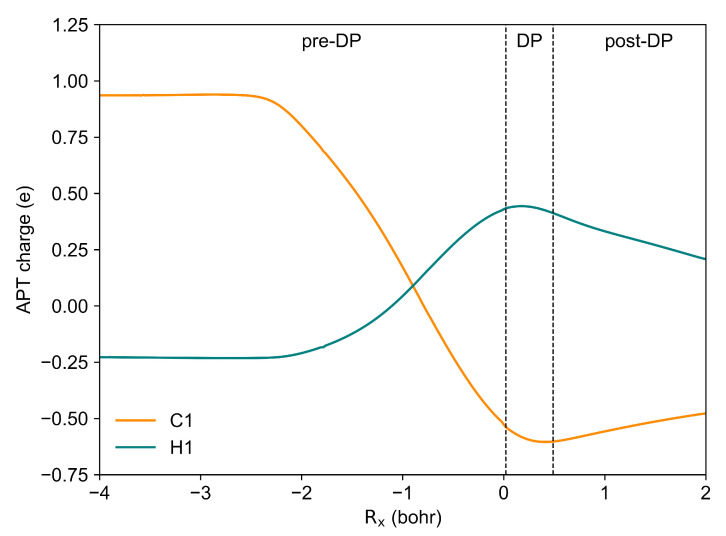
The evolution of APT charge of C1 and H1 atoms during the proton transfer in Lapworth’s cyanohydrin formation. The “DP” refers to the dressed proton state.

The formation of the Lapworth’s cyanohydrin is characterised by six ELF field catastrophes. This conclusion can also be derived from the six peaks in the Hausdorff distance where dH > 0.2. The first peak corresponds to an annihilation of $V_{1}$(C8,N) and $V_{2}$(C8,N) into a single V(C8,N) basin. Following this, the second and third peaks correspond to the annihilation and recreation of the $V_{2}$(O1) basin. These reorganisations of the valence electron density do not have a simple chemical interpretation. The fourth peak is related to the breaking of the C1-H1 bond. This leads to the formation of a proton surrounded by electron density, known as a dressed proton, in the form of the V(H1) basin and the non-bonding V(C1) basin in the vicinity of the C1 atom. The fifth and sixth peaks correspond to the creation of the $V_{3}$(O1) attractor in the valence shell of the O1 atom and the formation of the O1-H1 bond from $V_{3}$(O1) and V(H1) basins. A topological analysis of the ELF for the post-reaction complex revealed a flux of electron density from the $V_{1}$(O1) and $V_{2}$(O1) basins to the V(O1,H1) basin. The V(C1) basin is now described by two $V_{1,2}$(C1) basins with populations of 0.55e and 0.45e, respectively. These results align well with the APT charge data, as shown in Figure 5. In the metastable Lapworth’s cyanohydrin formation, the C1 atom initially exhibits a positive charge, which shifts to negative values as the proton transfer reaction progresses. This charge inversion on the C1 atom is indicative of electron density redistribution that stabilises the reaction intermediate. Conversely, the H1 atom starts with a negative partial charge, which reverses to a positive charge during the course of the reaction. This reversal signifies a transition in the electron density environment around the H1 atom, where it gains a partial positive character as it approaches the dressed proton state.

The formation of the C1-C15 bond in the TS3 is characterised by the occurrence of two ELF catastrophes. This is in good agreement with the calculated Hausdorff distances. The first peak, observed at $R_{x}$ = −1.620 Bohr, corresponds to the ELF topological catastrophe, where the V(C15) basin is created in association with the (3,−1) CP. The second peak corresponds to the formation of the C1-C15 bond, as evidenced by the annihilation of the V(C15) and V(C1) basins, along with the corresponding (3,−1) CP. This results in the creation of the V(C1,C15) basin, which is populated with a density of 1.08e. Notably, the population of the V(C1,C15) basin reaches a final value of 1.76e at the last step of the IRC path.

The proton transfer between the O1 and O2 atoms has been observed in TS4. The analysis of the Hausdorff distances, as depicted in Figure 4, reveals the presence of five peaks associated with five ELF catastrophes. The first peak, observed at $R_{x}$ = −0.330 Bohr, corresponds to the annihilation of the V(H1,O1) basin and results in the creation of the dressed proton V(H1) and monosynaptic V(O1) basins. The basin population of V(H1) after the catastrophe is 0.43e. The second and third peaks do not have a straightforward chemical interpretation. The $V_{3}$(O2) basin is created at $R_{x}$ = −0.210 Bohr, and the $V_{2}$(C8,N) basin is annihilated and merges with $V_{1}$(C8,N) to form a single V(C8,N) basin with a population of 4.35e. These ELF changes are also reflected by changes in the number of (3,−1) CPs. In the fourth catastrophe, the $V_{3}$(O2) and V(H1) basins are annihilated, resulting in the creation of the V(H1,O2) basin. At this point, the O2-H1 bond is represented by the V(H1,O2) basin with a population of 1.59e. The final peak corresponds to the annihilation of the third $V_{3}$(O1) basin.

The elimination of the cyanide group via TS5 is characterised by four ELF field catastrophes in the final stages. The first peak, observed in both dH(A,B) and dH(B,A), corresponds to a displacement of the V(O1) attractor into a new region without any significant ELF changes. The second peak results in the annihilation of the V(C1,C8) basin and the creation of the V(C1) and V(C8) basins. This is followed by an annihilation of the V(C1) basin. In the final catastrophe, the V(C8,N) basin is split into two $V_{1,2}$(C8,N) basins with an overall population of 3.53e.

In summary, the analysis of IRC path has revealed multiple ELF catastrophes at various stages of the chemical reactions studied. The Hausdorff distances and basin populations obtained from the analysis were found to be consistent with the proposed Lapworth mechanism. The ELF topology of the reaction was found to be in good agreement with the mechanistic understanding of the umpolung reaction.

## 3. Materials and Methods

The calculations for this study were performed in the gas phase, at 0 K, using the DFT method with the B3LYP electron density functional [31,32] as implemented in the Gaussian16 (G16) program (version C.01) [33]. The electron wave functions were described using the Pople basis set, 6-311++G(d,p) [34,35]. The optimisation of molecular geometries was performed using the Berny algorithm and obtained structures were verified by the calculation of infrared spectra to confirm its character on the potential energy hypersurface (PES). The presence of one imaginary frequency for transition states (TSs) and zero for reagents and products indicates that the stationary points correspond to the expected states.

The reaction path was calculated using IRC [36,37] implemented in G16. The initial Hessian matrix was calculated and recalculated every five steps. The correct localisation of TSs on PES was verified through an analysis of IRC paths and the subsequent optimisation of molecular structures to energy minima. These steps ensure that the results obtained are consistent with the expected reaction mechanism. The Gibbs free energies of activation, $\Delta G_{a}\left(I\right)$, and $\Delta G_{a}\left(\mathrm{II}\right)$, have been calculated as the difference between the sum of electronic and thermal free energies between TS and reagents and products, respectively. The Gibbs free energy of reaction, $\Delta G_{r}$, has been calculated as the difference between the sum of electronic and thermal free energies between products and reagents. The $\Delta G_{a}\left(I,\mathrm{II}\right)$ and $\Delta G_{r}$ values were calculated for a temperature of 298.15 K and pressure of 1 atm.

The evolution of chemical bonds was studied by comparing the topology of ELF for electronic structures of molecules localised at all points on IRC paths. Due to the flat character of PES, the gradient magnitude for the damped velocity Verlet (DVV) stopping criteria was turned off using the IOp(1/108 = −1) keyword in G16. Single-point calculations at the DFT(B3LYP)/6-311++G(d,p) computational level were performed to obtain a set of molecular orbitals for each molecular structure.

The topological analysis of ELF was performed using TopMod09 and MultiWFN programs [38,39]. A parallelepiped grid of points with a step size of 0.05 Bohr was used. The wave functions for the minima on PES and each structure on the IRC path were obtained by single-point DFT calculations at the DFT(B3LYP)/6-311++G(d,p) computational level. The analysis of catastrophes of the ELF field was performed using the MultiWFN and AyudaTop programs [39,40].

The Hausdorff distances [24], dH(A,B) and dH(B,A), were calculated for two sets of coordinates for the attractors of the ELF field. The A set contains the coordinates of all attractors for the electronic structure of molecule A on the IRC path preceding point B. The use of Hausdorff distances in the topological analysis of ELF in the context of reaction mechanisms has been previously studied by us and has been found to be an effective tool to investigate topological catastrophes in the evolution of chemical bonds [25]. This approach allows for a quantitative evaluation of the changes in the topology of the electron density during the reaction, providing a deeper understanding of the reaction mechanism.

## 4. Conclusions

The mechanism of the cyanide-type umpolung reaction in benzoin condensation has been investigated in detail by utilising the modern topological analysis of ELF and catastrophe theory. By applying this approach, a comprehensive understanding of the evolution of chemical bonds and non-bonding electron density in the reaction of benzaldehyde and cyanide ions was achieved. The use of the topological analysis of ELF and BET proved to be an effective method for characterising the mechanisms of this type of chemical reaction. The results may be summarised as follows:

1. The umpolung mechanism in the gas phase and without solvent assistance proceeds through five transition state structures. The formation of Lapworth’s cyanohydrin is not spontaneous, as evidenced by the positive $\Delta G_{r}$ value. Among these steps, the formation of the Lapworth’s cyanohydrin has the highest $\Delta G_{a}\left(I\right)$ value, making it the rate-determining step. Additionally, it was observed that the proton transfer in TS4 does not occur simultaneously with C-C bond formation and has a barrier-less character, as indicated by the negative $\Delta G_{a}\left(I\right)$ value.
2. The evolution of the electron localisation function (ELF) field in the studied umpolung mechanism is characterised by 17 distinct catastrophes. While some changes within the ELF field lack straightforward chemical interpretation, the application of topological analysis alongside catastrophe theory has enabled a detailed mapping of electron density evolution. In particular, a comprehensive analysis of ELF catastrophes in the cyanide-type umpolung reaction reveals that the formation of the C1-C8 bond occurs through a shift in the synapticity of the V(C8) basin to a V(C1,C8) basin at a distance of 2.029 Å. This was followed by proton transfer, resulting in the formation of Lapworth’s cyanohydrin and the appearance of additional electron density near the C1 atom, as indicated by the V(C1) basin. The formation of the C1-C15 bond in TS3 is characterised by two distinct ELF catastrophes: one describing the creation of non-bonded electron density near the C15 atom and the other describing the formation of the C1-C15 bond through the annihilation of the V(C1) and V(C15) basins. The proton transfer between the O1 and O2 atoms is found to involve the creation of a dressed-atom-type V(H1) basin. Finally, the dissociation of the C1-C8 bond and the elimination of the cyanide group occur through the annihilation of the disynaptic V(C1,C8) basin and the creation of monosynaptic V(C1) and V(C8) basins.
3. The applications of topological analysis of ELF and catastrophe theory confirm the well-established Lapworth mechanism for this reaction.

## Figures and Tables

**Table 1:** The values (kcal/mol) of the Gibbs free energy of activation, $\Delta G_{a}\left(I,\mathrm{II}\right)$, and the Gibbs free energy of reaction, $\Delta G_{r}$, calculated at DFT(B3LYP)/6-311++G(d,p) level.

Energy	TS1	TS2	TS3	TS4	TS5
** $\Delta G_{a}\left(I\right)$ **	10.88	43.03	1.48	−1.04	7.60
** $\Delta G_{a}\left(\mathrm{II}\right)$ **	4.17	40.53	0.26	0.05	17.77
** $\Delta G_{r}$ **	6.71	2.50	1.22	−1.09	−10.17

**Table 2:** The values (kcal/mol) of the Gibbs free energy of activation, $\Delta G_{a}\left(I,\mathrm{II}\right)$, and the Gibbs free energy of reaction, $\Delta G_{r}$, for the fourth umpolung reaction step.

Energy	B3LYP	M06-2X	BHHLYP	wB97XD	PBE0
6-31+G(d,p)					
** $\Delta G_{a}\left(I\right)$ **	−1.16	−1.15	−1.02	−1.36	−1.08
** $\Delta G_{a}\left(\mathrm{II}\right)$ **	−0.31	0.15	1.32	0.55	−0.40
** $\Delta G_{r}$ **	−0.85	−1.30	−2.34	−1.91	−0.68
6-311++G(d,p)					
** $\Delta G_{a}\left(I\right)$ **	−1.04	−1.10	−0.71	−1.01	−1.20
** $\Delta G_{a}\left(\mathrm{II}\right)$ **	0.05	0.68	1.72	0.77	−0.27
** $\Delta G_{r}$ **	−1.09	−1.78	−2.43	−1.78	−0.93
6-311++G(2df,p)					
** $\Delta G_{a}\left(I\right)$ **	−0.93	−1.14	−0.86	−1.16	−0.86
** $\Delta G_{a}\left(\mathrm{II}\right)$ **	−0.30	0.39	1.38	0.60	1.38
** $\Delta G_{r}$ **	−0.63	0.75	−2.24	−1.76	−2.24

## Data Availability

The raw data supporting the conclusions of this article will be made available by the authors on request.
